# A comparison study of monoexponential and fractional order calculus diffusion models and ^18^F-FDG PET in differentiating benign and malignant solitary pulmonary lesions and their pathological types

**DOI:** 10.3389/fonc.2022.907860

**Published:** 2022-07-21

**Authors:** Yu Luo, Han Jiang, Nan Meng, Zhun Huang, Ziqiang Li, Pengyang Feng, Ting Fang, Fangfang Fu, Jianmin Yuan, Zhe Wang, Yang Yang, Meiyun Wang

**Affiliations:** ^1^ Department of Medical Imaging, Zhengzhou University People’s Hospital & Henan Provincial People’s Hospital, Zhengzhou, China; ^2^ Academy of Medical Sciences, Zhengzhou University, Zhengzhou, China; ^3^ Department of Medical Imaging, Xinxiang Medical University & Henan Provincial People’s Hospital, Xinxiang, Henan, China; ^4^ Department of Medical Imaging, Henan University People’s Hospital & Henan Provincial People’s Hospital, Zhengzhou, China; ^5^ Central Research Institute, United Imaging Healthcare Group, Shanghai, China; ^6^ Beijing United Imaging Research Institute of Intelligent Imaging, Beijing, China

**Keywords:** solitary pulmonary lesions, lung cancer, PET/MR, diffusion-weighted imaging, fractional order calculus, differentiation diagnosis

## Abstract

**Objective:**

To evaluate the application value of monoexponential, fractional order calculus (FROC) diffusion models and PET imaging to distinguish between benign and malignant solitary pulmonary lesions (SPLs) and malignant SPLs with different pathological types and explore the correlation between each parameter and Ki67 expression.

**Methods:**

A total of 112 patients were enrolled in this study. Prior to treatment, all patients underwent a dedicated thoracic ^18^F-FDG PET/MR examination. Five parameters [including apparent diffusion coefficient (ADC) derived from the monoexponential model; diffusion coefficient (D), a microstructural quantity (μ), and fractional order parameter (β) derived from the FROC model and maximum standardized uptake value (SUVmax) derived from PET] were compared between benign and malignant SPLs and different pathological types of malignant SPLs. Independent sample t test, Mann-Whitney U test, DeLong test and receiver operating characteristic (ROC) curve analysis were used for statistical evaluation. Pearson correlation analysis was used to calculate the correlations between Ki-67 and ADC, D, μ, β, and SUVmax.

**Results:**

The ADC and D values were significantly higher and the μ and SUVmax values were significantly lower in the benign group [1.57 (1.37, 2.05) μm^2^/ms, 1.59 (1.52, 1.72) μm^2^/ms, 5.06 (3.76, 5.66) μm, 5.15 ± 2.60] than in the malignant group [1.32 (1.03, 1.51) μm^2^/ms, 1.43 (1.29, 1.52) μm^2^/ms, 7.06 (5.87, 9.45) μm, 9.85 ± 4.95]. The ADC, D and β values were significantly lower and the μ and SUVmax values were significantly higher in the squamous cell carcinoma (SCC) group [1.29 (0.66, 1.42) μm^2^/ms, 1.32 (1.02, 1.42) μm^2^/ms, 0.63 ± 0.10, 9.40 (7.76, 15.38) μm, 11.70 ± 5.98] than in the adenocarcinoma (AC) group [1.40 (1.28, 1.67) μm^2^/ms, 1.52 (1.44, 1.64) μm^2^/ms, 0.70 ± 0.10, 5.99 (4.54, 6.87) μm, 8.76 ± 4.18]. ROC curve analysis showed that for a single parameter, μ exhibited the best AUC value in discriminating between benign and malignant SPLs groups and AC and SCC groups (AUC = 0.824 and 0.911, respectively). Importantly, the combination of monoexponential, FROC models and PET imaging can further improve diagnostic performance (AUC = 0.872 and 0.922, respectively). The Pearson correlation analysis showed that Ki67 was positively correlated with μ value and negatively correlated with ADC and D values (r = 0.402, -0.346, -0.450, respectively).

**Conclusion:**

The parameters D and μ derived from the FROC model were superior to ADC and SUVmax in distinguishing benign from malignant SPLs and adenocarcinoma from squamous cell carcinoma, in addition, the combination of multiple parameters can further improve diagnostic performance. The non-Gaussian FROC diffusion model is expected to become a noninvasive quantitative imaging technique for identifying SPLs.

## Introduction

Lung cancer is the most common malignant tumor worldwide and ranks first in cancer-related deaths (1). Adenocarcinoma (AC) and squamous cell carcinoma (SCC) are the most common pathological types. Due to the lack of specificity of clinical manifestations, nearly 70% of lung cancer patients have already developed locally advanced or metastatic disease at the time of diagnosis (2). Ki-67 is an important marker of cell proliferation closely related to the occurrence and development of tumors. The pathological type of the tumor and the Ki67 index influence the treatment plan, treatment response and prognosis of lung cancer patients (3–6). Currently, the gold standard for the clinical diagnosis of lung cancer is histopathological biopsy. However, the clinical application of invasive procedure is limited due to the disadvantages of trauma, poor patient compliance, inability to obtain the overall situation and a high incidence of complications (7). Therefore, noninvasive diagnostic imaging of tumors is needed to overcome these limitations.

^18^F-FDG PET/CT provides metabolic and anatomical information and plays an indispensable role in the management of lung cancer patients. PET/MR is the organic integration of PET and MRI. Compared with PET/CT, PET/MR has the advantages of high soft-tissue resolution and markedly reduced radiation exposure while acquiring metabolic information. In addition, multiparameter magnetic resonance imaging can obtain additional information about tumor tissue, which probably helps improve the screening diagnosis of lung cancer (8). The maximum standardized uptake value (SUVmax) is a semiquantitative metabolic index commonly used in PET to reflect disease activity and invasiveness. Studies have shown that SUVmax can differentiate benign and malignant pulmonary nodules and pathological types of lung cancer (9, 10).

Diffusion-weighted magnetic resonance imaging (DWI) is a noninvasive imaging technique that can obtain information about the microscopic motion of water molecules and reflect changes at the cellular level (11). In DWI, the apparent diffusion coefficient (ADC) value quantifies the diffusion capacity of water molecules, providing information about cell density and membrane integrity (12). ADC has been used to distinguish between benign and malignant lung lesions (13). However, studies have shown that ADC values overlap in different pathological types of lung cancer; thus, ADC cannot effectively distinguish between AC and SCC (14). The conventional DWI is calculated using a monoexponential model, which assumes that the probability function of water molecule displacement follows a Gaussian distribution (15). The ADC value reflects the average diffusion coefficient of various tissue components, but in fact, due to the complexity of tissue structure, the diffusion behavior of water in tissue cells is more complicated than that of freely moving water (16). Increasing evidence shows that monoexponential DWI has nonnegligible limitations for evaluating the complex microstructure of tissues (17). Recognizing the limitations of ADC, Zhou et al. first proposed the fractional order calculus (FROC) model in 2010, which provides three parameters: diffusion coefficient (D), a spatial parameter (μ) and fractional order derivative in space (β) (18). These parameters can reflect not only the diffusion process but also the diffusion environment (μ) and structural complexity (β), thus making up for the shortcoming of the monoexponential model. The complexity of the internal microstructure of tumor tissues has been further revealed by using these parameters alone or in combination for the identification of tumor types, the staging of chronic hepatitis B liver fibrosis, the Lauren classification of gastric adenocarcinoma, assessing the response of gastrointestinal stromal tumors to targeted drugs, and preoperatively evaluating liver cancer (19–23).

To the best of our knowledge, the FROC model has not been used to assess lung diseases. Thus, the purpose of this study was to investigate the clinical utility of the FROC diffusion model in identifying SPLs, and whether the FROC model has advantages over the monoexponential model and PET imaging. Furthermore, we analyzed the correlation between each derived parameter and the Ki-67 index, thus guiding diagnostic and treatment strategies.

## Methods

### Study population

This prospective study was approved by the Ethics Committee of the local institution. Written informed consent was obtained from each patient. A total of 131 patients with suspected lung tumors without contraindications to PET/MR scans were examined from July 2020 to January 2022. The inclusion criteria were as follows: 1) chest CT showing that the maximum diameter of the lung tumor was ≥ 1.0 cm; 2) no patients received any related treatment; 3) no contraindications to MRI, such as ferromagnetic implants, claustrophobia; 4) primary lung cancer and benign lesions were confirmed by pathology (such as by surgery, needle biopsy, or bronchoscopy); and 5) Ki-67 expression detected by immunohistochemistry (IHC). The exclusion criteria were 1) lack of histopathological confirmation (n=6); 2) the quality of MRI images was poor, and the lesions were not clearly displayed (n=8); and 3) the imaging sequence was incomplete (n=5). In all, 112 patients were included in our study.

### Image acquisition

MRI and PET imaging were performed using an integrated 3.0T PET/MR (uPMR790, UIH) with a 12-channel phased-array body coil. The tracer used was ^18^F-FDG. Patients were required to fast for at least 6 hours to ensure that fasting blood glucose was below 8.0mmol/L. ^18^F-FDG was injected intravenously at a dose of 4.07MBq/kg, Whole lung images were collected after 1 h. Each patient was instructed to breathe normally in a supine position and was scanned from lung tip to diaphragmatic angle. Abdominal bands were used to reduce respiratory motion artifacts. PET reconstruction was performed using the iterative ordered subset expectation maximization algorithm (OSEM), and Dixon MRI sequence attenuation was used to correct gamma rays. The imaging protocol included PET imaging, MR-based attenuation correction (2.04min), T2-weighted imaging (2.26min), T1-weighted imaging (14S), and multi-b-values DWI sequence(5.15min). The monoexponential model was performed by using two b values (0, 800 s/mm^2^). The FROC model was performed by using twelve b values (0, 25, 50, 100, 150, 200, 400, 600, 800, 1000, 1500, 2000 s/mm^2^), separation between two diffusion gradient lobes, Δ = 23.96 msec; duration of each diffusion gradient, δ = 17.36 msec. MRI acquisition parameters are shown in **Table 1**.

**Table 1:** Scanning parameters of MRI.

	T_2_WI	T_1_WI	Multiple b-value DWI
TR (ms)	3315	4.24	1620
TE (ms)	90.2	1.13	69.6
Slice thickness (mm)	5	6	5
Gap (mm)	20	0	20
FOV (mm)	400×300	400×300	400×300
Matrix	320×70	320×70	128×100
NEX	2	1	1, 1, 2, 2, 4, 4, 6, 6, 8, 10
b-values	–	–	0, 25, 50, 100, 150, 200, 400, 600, 800, 1000, 1500, 2000s/mm^2^
Orientation	Axia	Axia	Axia
Breath control	Breathing navigation	Breath-holding	Breathe freely
Scanning time	2.26min	14s	5.15min

DWI, diffusion-weighted imaging; FOV, field of view; NEX, number of excitations; T1WI, T1 weighted imaging; T2WI, T2 weighted imaging; TE, echo time; TR, repetition time.

### Image processing

All images were imported into the United imaging workstation (uWS-MR, UIH, Shanghai, China) for postprocessing. Two radiologists (with 5 and 10 years of work experience, respectively) independently measured the SUVmax value. The PET/MR postprocessing software automatically delineated the volumes of interest (VOIs) of the lesions and calculated the SUVmax at a 40% relative threshold. The regions of interest (ROIs) were manually plotted independently by two radiologists. Before drawing ROIs, both radiologists were blinded to the pathological results and clinical data, cystic components, necrotic areas, hemorrhage, and calcification were avoided by referring to T1- and T2-weighted images. The ROIs were plotted on DWI images along the inner edge of the solid tumor region and then propagated to the corresponding μ, D, β and ADC maps. The average value measured by the two radiologists was calculated for final analyses. DWI and FROC image processing and analysis were performed using custom software developed in MATLAB.

### Image analysis

The formulas used to calculate ADC values and FROC model parameters are as follows:

(1) Monoexponential model: The parameter map of monoexponential DWI was calculated by the following fitting formula (22):


$${\text{S}}_{\text{b}}{\text{/S}}_{0}=\text{exp}\left(\text{-b}\times ADC\right)$$

where S_0_ and S_b_ are respectively the signal intensity when b values of 0 and 800 s/mm^2^ are applied. ADC (μm^2^/ms) is the abbreviation of the apparent diffusion coefficient.

(2) Fractional-order calculus model: The parameter maps of FROC-DWI were calculated by the following fitting formula (21, 24):


$${\text{S}}_{\text{b}}{\text{/S}}_{0}=\text{exp}\left[\text{-D}\mu^{2(\beta -1)}{\left(\gamma \text{Gd}\delta \right)}^{2\beta }(\Delta -\frac{2\beta -1}{2\beta +1}\delta )\right]$$

where D (μm^2^/ms) is the diffusion coefficient, which is similar to ADC, β (dimensionless between 0 and 1) represents the fractional order derivative in space and reflects the uniformity of the internal structure of tissue, μ(μm) is a spatial parameter reflecting the diffusion distance of water molecules, which is a unique parameter in the FROC model, G_d_ is the diffusion gradient amplitude, δ is the diffusion gradient pulse width, Δ is the gradient lobe separation, and γ is the spin ratio. The 12 b-values diffusion images were fitted to the FROC model voxel-by-voxel by using a nonnegative least square fitting algorithm to generate the three parameter maps.

### Histopathologic analysis

All specimens were embedded in paraffin, sectioned, and stained with conventional HE, and the expression of Ki-67 was analyzed by immunohistochemistry using mouse anti-human Ki-67 monoclonal antibody (MIB-1, DAKO). The pathological types of the patients were diagnosed by a senior pathologist blinded to the clinical and imaging data. The positive expression of Ki67 was in the nucleus and stained into brownish-yellow granules. The percentage of positive tumor cells (%) was counted and recorded.

### Statistical analysis

All statistical analyses and plots were performed using SPSS(version 26.0), MedCalc(version 15.2.2), and GraphPad Prism(version 8.0). A two-sided P value of less than 0.05 was considered to indicate statistical significance. The intraclass correlation coefficient (ICC) was used to assess the agreement of the measured parameters between the two radiologists, and interpreted as follows: poor agreement 0.00 - 0.20, fair agreement 0.21 - 0.40, moderate agreement 0.41 - 0.60, good agreement 0.61 - 0.80, and excellent agreement 0.81 - 1.00. All parameters were tested for normal distribution using the Kolmogorov-Smirnov test, data conforming to a normal distribution were expressed as the mean ± standard deviation, and data not conforming to a normal distribution were expressed as median and interquartile range. Independent sample t-tests (normal distribution) and Mann-Whitney U tests (nonnormal distribution) were used to compare the differences in the parameter values between the benign and malignant groups and between malignant SPLs with different pathological types. The correlation between each parameter and the Ki-67 index was analyzed by Pearson correlation. FROC parameters were combined using the logistic regression expression: P_0_ = exp (a_0_ + a_1_D + a_2_β + a_3_μ)/[1 + exp (a_0_ + a_1_D + a_2_β + a_3_μ)], where a_0_ is a constant, and a_i_ (for i = 1, 2, 3) is the regression coefficient of D, β, and μ, respectively. The regression coefficients were estimated by using a maximum likelihood method (19, 23). The area under the curve (AUC) given by the receiver operating characteristic (ROC) curve was used to evaluate the ability of different parameters to distinguish benign and malignant SPLs and different pathological types: (0.50, 0.70) indicated low diagnostic value, (0.70, 0.90) indicated moderate diagnostic value, and above 0.90 indicated high diagnostic value (25). The optimal cutoff value was selected according to the maximum value of the Youden Index and the corresponding accuracy, sensitivity and specificity values were calculated. The DeLong test was used for the statistical comparison of AUCs with different parameters.

## Results

### Demographics

**Figure 1:**
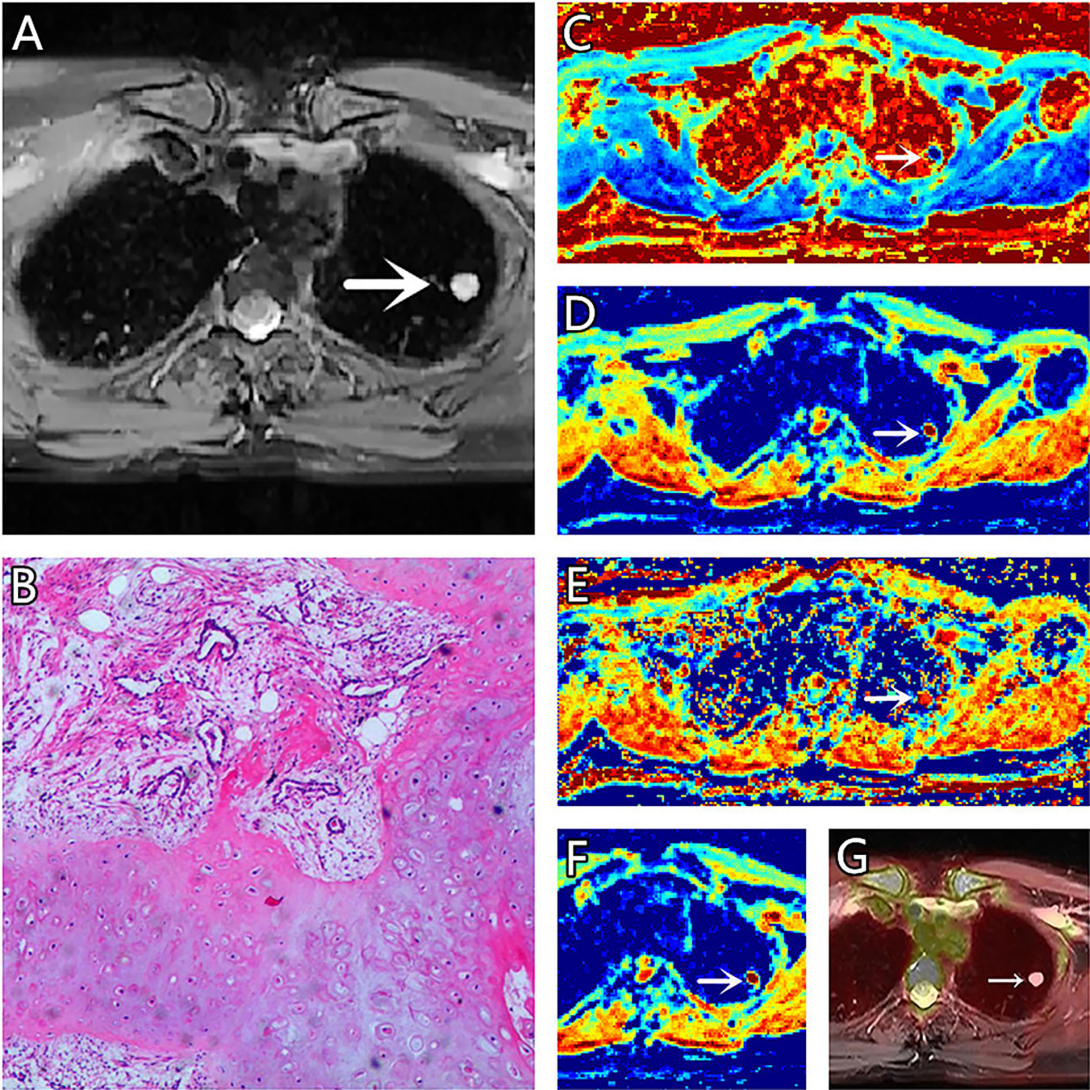
**(A–G)** A 51-year-old female patient, with hamartoma in the left lung, **(A)** is T2 weighted anatomic image, **(B)** is hematoxylin and eosin (H&E) staining image which confirms this lesion to be hamartoma, **(C)** is μ map with μ= 3.21 μm, **(D)** is D map with D = 1.73 μm^2^/ms, **(E)** is β map with β= 0.78, **(F)** is ADC map with ADC = 1.99 μm^2^/ms, **(G)** is the fusion image of the SUV map and the attenuation correction map with SUVmax = 0.5.

**Figure 2:**
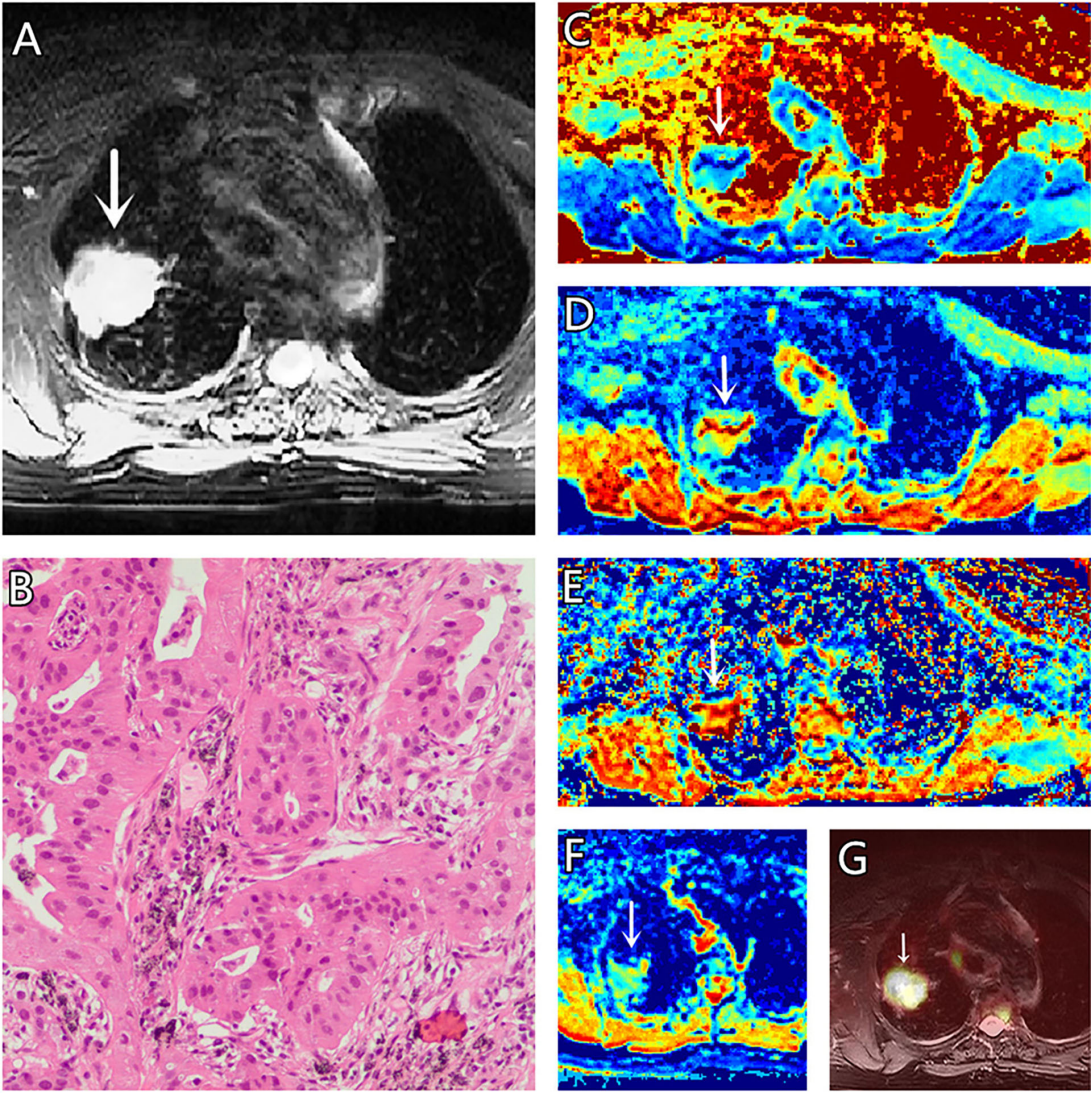
**(A–G)** A 73-year-old female patient, with adenocarcinoma **(AC)** in the right lung, **(A)** is T2 weighted anatomic image, **(B)** is hematoxylin and eosin (H&E) staining image which confirms this lesion to be AC, **(C)** is μ map with μ= 4.77 μm, **(D)** is D map with D = 1.69 μm^2^/ms, **(E)** is β map with β = 0.83, **(F)** is ADC map with ADC = 1.37 μm^2^/ms, **(G)** is the fusion image of the SUV map and the attenuation correction map with SUVmax = 3.41.

**Figure 3:**
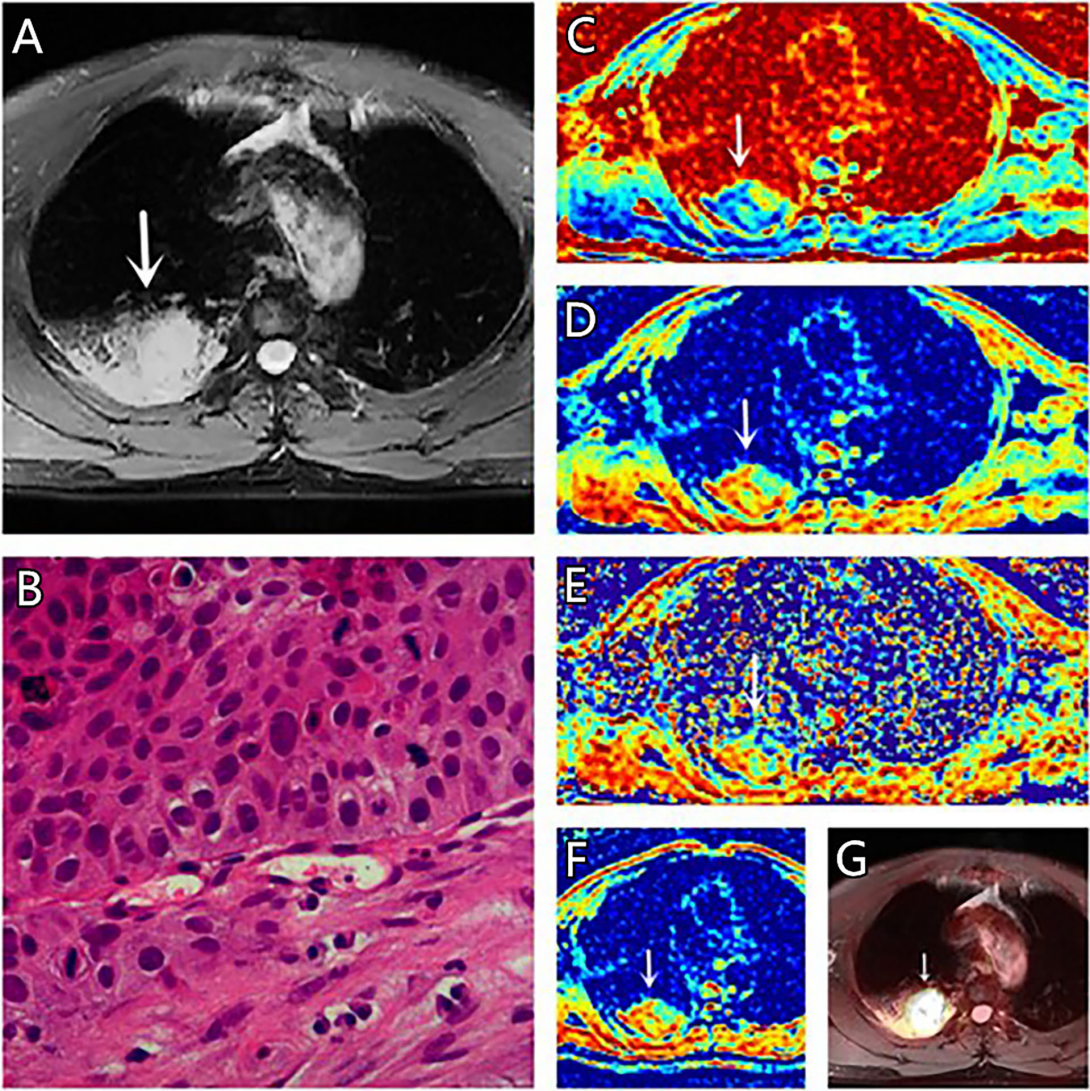
**(A–G)** A 67-year-old male patient, with squamous cell carcinoma (SCC) in the right lung, **(A)** is T2 weighted anatomic image, **(B)** is hematoxylin and eosin (H&E) staining image which confirms this lesion to be SCC, **(C)** is μ map with μ= 7.44 μm, **(D)** is D map with D = 1.42 μm^2^/ms, **(E)** is β map with β= 0.68, **(F)** is ADC map with ADC = 1.29 μm^2^/ms, **(G)** is the fusion image of the SUV map and the attenuation correction map with SUVmax = 18.75.

A total of 19 patients were excluded; as a result, 112 patients were included in this study. Pathological findings were used as the gold standard. 35 patients were diagnosed with benign lesions (25 males, 10 females; range, 40-81 years; median age 57 years; mean age 59 years), including focal pneumonia (N = 31), bronchial adenoma (N = 2), hamartoma (N = 1), and infectious granuloma (N = 1), and 77 patients were diagnosed with malignant lesions (45 males, 32 females; range, 33-79 years; median age 63 years; mean age 61 years), including AC (N = 40), SCC (N = 25), small cell carcinoma (N = 7), and lung metastasis (N = 4 come from gastrointestinal tumors and N = 1 come from Lymphoma) (**Table 2**, **Figures 1**–**3**).

**Table 2:** Demographics.

Characteristics	Malignant SPLs	Benign SPLs	AC	SCC
Number of patients	77	35	40	25
Gender (M:F)	45: 32	25: 10	21: 19	17: 8
Subtypes	AC (N = 40)	Focal pneumonia (N=31)		
	SCC (N = 25)	Bronchial adenoma (N=2)		
	Small cell carcinoma (N = 7)	Hamartoma (N=1)		
	Lung metastasis (N = 5)	Infectious granuloma (N=1)		

AC, adenocarcinoma; SCC, squamous cell carcinoma; SPLs, solitary pulmonary lesions.

### Consistency analysis

The measurements of all parameters by the two radiologists showed excellent agreement. The ICCs for ADC, μ, D, β, and SUVmax were 0.866, 0.903, 0.891, 0.848, and 0.861, respectively (**Table 3**).

**Table 3:** Mean and consistency analysis of parameters as respectively measured by both radiologists.

Parameters	Radiologist 1(Mean ± SD)	Radiologist 2 (Mean ± SD)	ICC	95% CI
ADC(μm^2^/ms)	1.43 ± 0.55	1.45 ± 0.54	0.866	0.811-0.906
μ(μm)	7.56 ± 4.68	7.37 ± 4.71	0.903	0.863-0.933
D(μm^2^/ms)	1.47 ± 0.24	1.46 ± 0.25	0.891	0.846-0.924
β	0.67 ± 0.10	0.68 ± 0.11	0.848	0.786-0.893
SUVmax	8.38 ± 4.88	8.38 ± 5.20	0.861	0.805-0.903

### Parameter comparison

**Figure 4:**
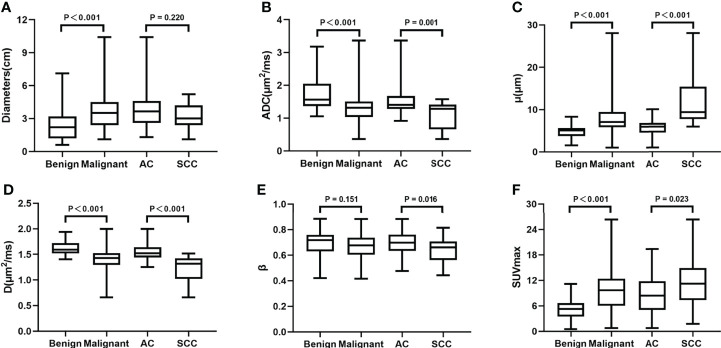
Boxplots of Diameters, ADC, μ, D, β and SUVmax between the benign and malignant SPLs groups, and between AC and SCC groups. ADC, apparent diffusion coefficient; D, diffusion coefficient; SCC, squamous cell carcinoma; SUVmax, maximum value of standard uptake value.

There was no significant difference in age between the benign and malignant SPLs group patients or between the AC and SCC group patients (P = 0.233 and 0.480, respectively). The diameter of the malignant SPLs group was significantly larger than that of the benign SPLs group (P<0.001); however, there was no significant difference between the AC group and the SCC group (P = 0.220). The μ and SUVmax values of benign SPLs were significantly lower, and the ADC and D values were significantly higher than the corresponding values in malignant SPLs. In distinguishing pathological types, μ and SUVmax values in the AC group were significantly lower, and ADC and D values were significantly higher than the corresponding values in the SCC group. The β value of AC was significantly higher than that of SCC(P = 0.016), however, there was no difference in β values between the benign and malignant groups (P = 0.151) (**Table 4**, **Figure 4**).

**Table 4:** Comparison of different parameters between the benign and malignant SPLs groups, and between AC and SCC groups.

Parameters	Benign	Malignant	P Value	AC	SCC	P Value
Age(years)	57(49, 70)	63(56, 68)	0.233^a^	60.45 ± 11.27	62.28 ± 7.85	0.480^b^
Diameters(cm)	2.20(1.20, 3.20)	3.50(2.40, 4.50)	<0.001^a^	3.70 ± 1.62	3.24 ± 1.19	0.220^b^
ADC(μm^2^/ms)	1.57(1.37, 2.05)	1.32(1.03, 1.51)	<0.001^a^	1.40(1.28, 1.67)	1.29(0.66, 1.42)	0.001^a^
μ(μm)	5.06(3.76, 5.66)	7.06(5.87, 9.45)	<0.001^a^	5.99(4.54, 6.87)	9.40(7.76, 15.38)	<0.001^a^
D(μm^2^/ms)	1.59(1.52, 1.72)	1.43(1.29, 1.52)	<0.001^a^	1.52(1.44, 1.64)	1.32(1.02, 1.42)	<0.001^a^
β	0.72(0.63, 0.76)	0.68(0.60, 0.74)	0.151^a^	0.70 ± 0.10	0.63 ± 0.10	0.016^b^
SUVmax	5.15 ± 2.60	9.85 ± 4.95	<0.001^b^	8.76 ± 4.18	11.70 ± 5.98	0.023^b^

Data were expressed as mean ± standard deviation (SD) or median (first quartile, third quartile) depending on whether the normal distribution is being followed.

AC, adenocarcinoma; SCC, squamous cell carcinoma.

P < 0.05 indicates that the difference is statistically significant.

a Comparisons were performed by Mann–Whitney U test.

b Comparisons were performed by independent sample t-test.

### ROC analysis

**Figure 5:**
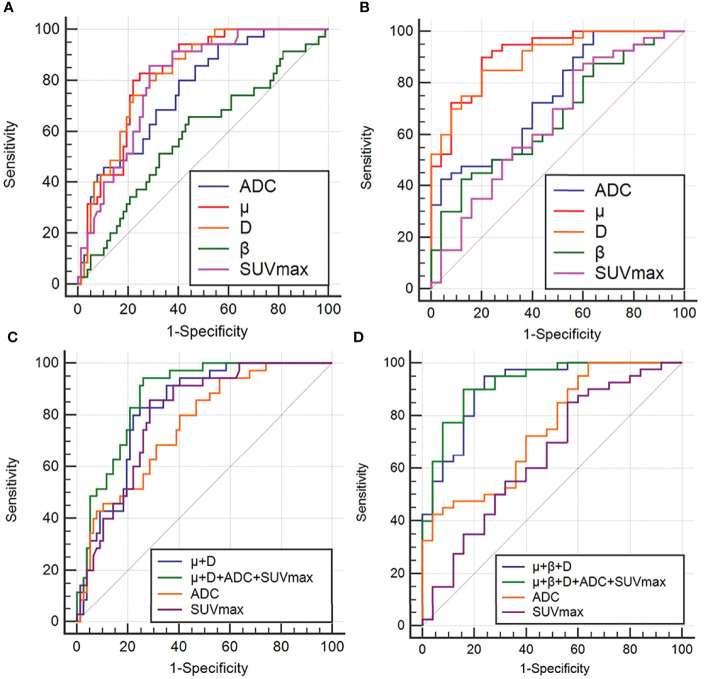
ROC curves of ADC, μ, D, β, SUVmax and different combinations of μ, D, β, ADC and SUVmax to distinguish between benign and malignant groups **(A, C)**, and between AC and SCC groups **(B, D)**. ROC analysis for differentiation of malignant and benign groups **(A, C)**; ROC analysis for differentiation of adenocarcinoma (AC) and squamous cell carcinoma (SCC) groups**(B, D)**.

Regarding the identification of benign and malignant SPLs, AUC(μ) > AUC(D) > AUC (SUVmax) > AUC(ADC) > AUC(β) (AUC = 0.824, 0.821, 0.801, 0.760, 0.585, respectively). The diagnostic efficiencies of different methods and their combinations were as follows: AUC (μ+D+ADC+SUVmax) > AUC (μ+D) > AUC (SUVmax) > AUC (ADC)(AUC = 0.872, 0.825, 0.801, 0.760, respectively). **Table 5** and **Figure 5** show the AUC, Youden index, accuracy, sensitivity, specificity, and 95% confidence interval of each parameter.

**Table 5:** ROC analysis of the diagnostic performance for different parameters and methods alone or in combination for distinguishing benign and malignant groups, and distinguishing AC and SCC groups.

Parameters	Cutoff	AUC	P Value	Youden index	Accuracy	Sensitivity	Specificity		95%CI
**Benign and Malignant groups**									
ADC(μm^2^/ms)	1.36	0.760	<0.0001	0.3974	74.1	80.00	59.74	0.670 - 0.836	
μ(μm)	5.85	0.824	<0.0001	0.5818	72.3	82.86	75.32	0.741 - 0.890	
D(μm^2^/ms)	1.53	0.821	<0.0001	0.5221	73.2	74.29	77.92	0.737 - 0.887	
β	0.69	0.585	0.1534	/	/	/	/	0.488 - 0.677	
SUVmax	6.85	0.801	<0.0001	0.5714	72.3	85.71	71.43	0.715 - 0.870	
μ + D	0.66	0.825	<0.0001	0.5818	71.4	82.86	75.32	0.742 - 0.890	
Combined Diagnosis^a^	0.79	0.872	<0.0001	0.6727	76.8	97.14	70.13	0.796 - 0.928	
**AC and SCC groups**									
ADC(μm^2^/ms)	1.51	0.747	<0.0001	0.3850	69.2	42.50	96.00	0.624 - 0.847	
μ(μm)	7.33	0.911	<0.0001	0.7000	83.1	90.00	80.00	0.814 - 0.967	
D(μm^2^/ms)	1.43	0.897	<0.0001	0.6500	80.0	85.00	80.00	0.796 - 0.959	
β	0.73	0.664	0.0162	0.3050	64.6	42.50	88.00	0.536 - 0.776	
SUVmax	12.36	0.648	0.0413	0.2900	66.2	85.00	44.00	0.520 - 0.762	
μ + β + D	0.58	0.908	<0.0001	0.7100	84.6	95.00	76.00	0.810 - 0.966	
Combined Diagnosis^b^	0.42	0.922	<0.0001	0.7400	86.2	90.00	84.00	0.828 - 0.974	

a The combined diagnosis represents μ + D + ADC + SUVmax.

b The combined diagnosis represents μ + β + D + ADC + SUVmax.

AUC, Area Under the Curve.

For AC and SCC, AUC (μ) > AUC (D) > AUC (ADC) > AUC (β) > AUC (SUVmax)(AUC = 0.911, 0.897, 0.747, 0.664, 0.648, respectively). The diagnostic efficiencies of different methods and their combinations were as follows: AUC (μ+β+D+ADC+SUVmax) > AUC (μ+β+D) > AUC (ADC) > AUC(SUVmax)(AUC = 0.922, 0.908, 0.747, 0.648, respectively). Detailed values and comparisons are presented in **Table 5** and **Figure 5**.

Comparison of AUC curves in the benign and malignant SPLs groups showed that D (area difference 0.0612; P = 0.0397) and the combined parameters (μ+D+ADC+SUVmax) (area difference 0.112; P = 0.0077) were significantly higher than the AUC of ADC, In addition, the combined parameter (μ+D+ADC+SUVmax) (area difference 0.0712; P = 0.0155) was significantly higher than the AUC of SUVmax (**Table 6**). Comparison of AUC curves in the AC and SCC groups showed that μ(area difference 0.164; P = 0.0011), D (area difference 0.150; P = 0.0012) and the combined parameters (μ+β+D) (area difference 0.161; P = 0.0016), (μ+β+D+ADC+SUVmax) (area difference 0.175; P = 0.0017) were significantly higher than the AUC of ADC, In addition, μ(area difference 0.263; P = 0.0008), D (area difference 0.249; P = 0.0012) and the combined parameters (μ+β+D) (area difference 0.260; P = 0.0013), (μ+β+D+ADC+SUVmax) (area difference 0.274; P = 0.0002) were significantly higher than the AUC of SUVmax (**Table 6**).

**Table 6:** The P value of ROC curve pairwise comparison of different parameters and different methods in the malignant and benign SPLs groups, and AC and SCC groups.

Parameters	Comparison with ADC	Comparison with SUVmax	Comparison with combined diagnosis
**Benign and Malignant groups**			
ADC(μm^2^/ms)	/	Z = 0.730, P = 0.4655	Z = 2.664, P = 0.0077
μ(μm)	Z = 1.897, P = 0.0579	Z = 0.443, P = 0.6576	Z = 1.512, P = 0.1305
D(μm^2^/ms)	Z = 2.057, P = 0.0397	Z = 0.391, P = 0.6962	Z = 1.547, P = 0.1219
SUVmax	Z = 0.730, P = 0.4655	/	Z = 2.420, P = 0.0155
μ + D	Z = 1.906, P = 0.0567	Z = 0.459, P = 0.6465	Z = 1.501, P = 0.1334
Combined Diagnosis^a^	Z = 2.664, P = 0.0077	Z = 2.420, P = 0.0155	/
**AC and SCC groups**			
ADC(μm^2^/ms)	/	Z = 1.161, P = 0.2457	Z = 3.139, P = 0.0017
μ(μm)	Z = 3.276, P = 0.0011	Z = 3.337, P = 0.0008	Z = 0.769, P = 0.4418
D(μm^2^/ms)	Z = 3.246, P = 0.0012	Z =3.250, P = 0.0012	Z = 1.116, P = 0.0155
β	Z =1.075, P = 0.2822	Z = 0.174, P = 0.8618	Z = 3.553, P = 0.0004
SUVmax	Z = 1.161, P = 0.2457	/	Z = 3.762, P = 0.0002
μ + β + D	Z = 3.154, P = 0.0016	Z = 3.217, P = 0.0013	Z = 0.889, P = 0.3739
Combined Diagnosis^b^	Z = 3.139, P = 0.0017	Z = 3.762, P = 0.0002	/


a The combined diagnosis represents μ + D + ADC + SUVmax.

b The combined diagnosis represents μ + β + D + ADC + SUVmax.

### Pearson’s correlation analysis

**Figure 6:**
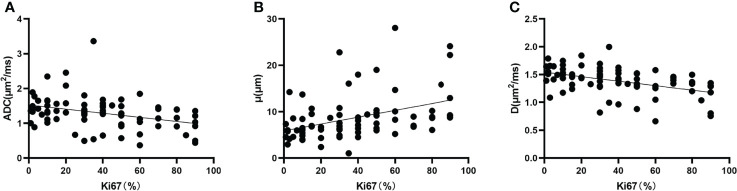
Correlation between **(A)** Ki67 and ADC (r = -0.346, P = 0.002), **(B)** Ki67 and μ (r = 0.402, P < 0.001), and **(C)** Ki67 and D (r = -0.450, P < 0.001). D, diffusion coefficient.

Ki67 was positively correlated with μ (r = 0.402, P < 0.001) and negatively correlated with ADC (r = -0.346, P = 0.002) and D (r = -0.450, P < 0.001) (**Figure 6**).

## Discussion

This study demonstrated the feasibility and validity of FROC model parameters (i.e., D, μ, β) in differentiating benign SPLs from malignant SPLs and AC from SCC. Compared to traditional monoexponential model and PET imaging, ROC analysis showed that the μ value had the highest diagnostic performance when comparing individual parameter. More importantly, the combination of FROC-derived parameters, ADC and SUVmax has better diagnostic performance and accuracy. The Pearson correlation analysis showed that Ki67 was positively correlated with μ and negatively correlated with ADC and D. The FROC model provides a new perspective to explore information about the tissue microenvironment and has great potential for noninvasive identification of SPLs.

The ADC value was significantly higher in the benign than in the malignant SPLs group, and AC than SCC. The D value was similar to the traditional diffusion coefficient (19) and can reflect the diffusion velocity of water molecules, and the result was consistent with the ADC value. This result may be because the density of cells in the solid part of malignant SPLs is higher than that of benign SPLs, the cancerous mesenchymal component is increased (26), the extracellular space is reduced, and the water molecule activity is restricted. The traditional ADC value averages the heterogeneity of the diffusion process. Under the influence of the complex tissue environment in the human body due to cell membranes, cell growth patterns, and nucleoplasmic ratios, the motion of water molecules deviates from a Gaussian distribution. The FROC model was fitted by multiple b-values to obtain a more accurate D value. The results of this study indicate that the D value has better differential diagnostic ability than the ADC value and might become a potential imaging marker.

μ is unique to the FROC diffusion model, which is related to the mean free length of water molecules (18, 27, 28). Chen et al. concluded that the μ value of Warthin tumors was higher than that of pleomorphic adenomas in a study of salivary gland tumors because the former was a salivary gland tumor characterized by hypercellularity and hypervascularity, resulting in a reduced mean free length of spread (29). In another study of clear cell renal carcinoma, high-grade tumors had significantly higher μ values than low-grade tumors, indicating the shortened mean free length in the former (30). This is consistent with our findings that μ values were significantly higher in malignant SPLs and SCC than in benign SPLs and AC. This finding can be attributed to the rapid proliferation of malignant tumor tissues, with increased cell numbers, higher cell density, and limited diffusion distance of water molecules, resulting in higher μ values in malignant SPLs. A previous study by Chen et al. revealed that the nucleoplasmic ratio of SCC was significantly higher than that of AC, the glandular lumen and cytoplasm of cells in AC contain mucous components, and the content of free water molecules in AC is higher than that in SCC (31). This pathological feature decreases the diffusion mean free length, resulting in a higher μ value in SCC than in AC. However, in the study of pediatric brain tumors by Sui et al, μ decreased from low-grade (less diffusion-restricted) to high-grade (more diffusion-restricted) brain tumors (19). These controversial results may be due to the heterogeneity of study subjects, including different scanning parameters, and different biological organs and tissues.

Previous studies have suggested that the β value reflects the uniformity of tissue structure and is inversely proportional to the complexity of the tissue microstructure (19, 32). A smaller β value indicates more significant intravoxel heterogeneity. In our study, the β value did not contribute significantly to differentiating benign and malignant SPLs, which may be related to the inclusion of other types of malignancies such as small cell lung cancer and metastatic lung cancer in the malignant SPLs group. The β value of the SCC group was significantly lower than that of the AC group, and this finding may be because the tumor tissue is inherently heterogeneous with complex internal components, and the tissue structure of SCC is more complex and heterogeneous than that of AC. These findings are consistent with the conclusions of Koyama et al. Koyama et al. found that AC tumor cells grew along the original cell wall in an alternative way, retaining the underlying normal structure, while SCC grew nonalternatively, with solid masses destroying the normal alveolar structure (33).

In our study, the SUVmax value was significantly higher in malignant than benign SPLs, and SCC had higher FDG uptake than AC in the comparison of different lung cancer pathological types, consistent with the previous findings by Fang et al (34). ^18^F-FDG PET imaging showed the metabolic activity of ^18^F-FDG in tumor cells, taking advantage of the fact that the anaerobic glycolysis metabolism of most malignant tumor cells is more vigorous than that of normal tissue cells (35). Smolle et al. found that tumor FDG uptake is associated with overexpression of the glucose transporter GLUT1 in lung cancer, and GLUT1 is highly expressed in SCC (36), which explains why the SUVmax of SCC is higher than that of AC. However, false positive results of increased FDG uptake can also occur during active inflammation, infection, or fibrosis. In addition, patient BMI, weight, blood glucose level, and time of imaging can also affect FDG uptake; therefore, the specificity of ^18^F-FDG PET examination is low.

Ki67 was expressed during cell proliferation but not in quiescent G0 cells, which was associated with proliferative activity and FDG uptake in lung cancer cells (37, 38). Our study showed that Ki67 was positively correlated with μ and negatively correlated with ADC and D, which is consistent with the results of Huang et al. (39). The explanation may be as follows: as the proliferation activity of tumor cells increased the cell density increased, the free diffusion of water molecules decreased, and the μ value increased, while the ADC and D values decreased correspondingly.

Our study has several limitations. First, the maximum b value used in the FROC analysis is different from that used in the monoexponential model, resulting in a different signal-to-noise ratio. Second, the degree of differentiation of malignant tumors was not studied, which may bias the results because of histological heterogeneity. Further large-cohort studies are needed to verify the value of FROC diffusion parameters in the differentiation study of malignant SPLs subgroups. Third, the sample size was relatively small. The calculation of AUC did not consider the influence of possible parameter overfitting. With a larger sample size, a training set and a validation set can be evaluated. Finally, despite the use of abdominal bands, it was difficult to avoid respiratory artifacts with PET/MR.

In conclusion, the FROC model reflects diffusion information (D) and provides parameters (μ and β) related to tumor heterogeneity, Our study demonstrated the feasibility of the FROC model for the noninvasive identification of SPLs, especially the D and μ values. The combined parameters of the FROC model can further improve the diagnostic performance compared to individual parameters. In addition, the combination of monoexponential, FROC models and PET imaging provided the best diagnostic performance and accuracy. The FROC model can provide rich information for intravoxel structural heterogeneity and better reflect the potential microstructural features of SPLs, which has great potential value in future clinical diagnosis and treatment.

## Data availability statement

The original contributions presented in the study are included in the article/supplementary material. Further inquiries can be directed to the corresponding author.

## Ethics statement

The studies involving human participants were reviewed and approved by ethics committee of Henan Provincial People’s Hospital. The patients/participants provided their written informed consent to participate in this study. Written informed consent was obtained from the individual(s) for the publication of any potentially identifiable images or data included in this article.

## Author contributions

Drafting of the manuscript: YL, NM, and HJ. Study concept and design: YL, JY, and ZW. Acquisition, analysis, or interpretation of data: YL, NM, HJ, ZH, ZL, PF, and TF. Critical revision of the manuscript for important intellectual content: YL, FF, ZW, JY, and YY. Statistical analysis: YL, NM, FF, and ZW. Study supervision: MW. All authors contributed to the article and approved the submitted version.

## Funding

This work was supported by the National Key R&D Program of China (2017YFE0103600), the Henan Medical Science and Technology Research Program (2018020357 and 2018020367), the National Natural Science Foundation of China (81720108021 and 31470047), Zhongyuan Thousand Talents Plan Project - Basic Research Leader Talent (ZYQR201810117), Zhengzhou Collaborative Innovation Major Project (20XTZX05015), Henan provincial science and technology research projects (212102310689) and Key Project of Henan Province Medical Science and Technology Project (LHGJ20210001).

## Conflict of interest

The authors JY and ZW were employed by United Imaging Healthcare Group, and the author YY was employed by Beijing United Imaging Research Institute of Intelligent Imaging.

The remaining authors declare that the research was conducted in the absence of any commercial or financial relationships that could be construed as a potential conflict of interest.

## Publisher’s note

All claims expressed in this article are solely those of the authors and do not necessarily represent those of their affiliated organizations, or those of the publisher, the editors and the reviewers. Any product that may be evaluated in this article, or claim that may be made by its manufacturer, is not guaranteed or endorsed by the publisher.

## Abbreviations

AC, adenocarcinoma; ADC, apparent diffusion coefficient; AUC, area under the curve; D, diffusion coefficient; DWI, diffusion-weighted magnetic resonance imaging; FROC, fractional order calculus; ROC, receiver operating characteristic; SCC, squamous cell carcinoma; SPLs, solitary pulmonary lesions; SUVmax, maximum standardized uptake value; β, fractional order derivative in space; μ, a spatial parameter.
